# The Outcome of Status Epilepticus Among Adults in Aseer Region of Saudi Arabia

**DOI:** 10.7759/cureus.22880

**Published:** 2022-03-06

**Authors:** Zubaidah S Alahmari, Hajr Almarie, Budoor Alahmari, Asiah Al Bin Abdullah, Shuruq M Al-Ayaffi, Velu M Murugan

**Affiliations:** 1 Internal Medicine and Neurology, King Khalid University, Abha, SAU; 2 Medicine and Neurology, King Khalid University, Abha, SAU; 3 College of Medicine and Surgery, King Khalid University, Aseer, SAU; 4 Medicine and Neurology, Aseer Central Hospital, Abha, SAU; 5 Neurology, Aseer Central Hospital, Abha, SAU

**Keywords:** epilepsy, status epilepticus, seizures, outcome, risk factors, mortality, adult, clinical complications

## Abstract

Introduction

Epileptic seizure episodes can vary from brief and nearly undetectable to long periods of vigorous shaking. These episodes can result in physical injuries, occasionally including broken bones. With epilepsy, seizures tend to recur and, as a rule, have no immediate underlying cause. Status epilepticus (SE) is an attack of a seizure lasting for more than five minutes or two or more seizures without the person returning to normal between the attacks. Previous definitions used a 30-minute time limit. This study aimed to assess the clinical outcome of SE among adult patients in the Aseer region.

Materials and methods

A retrospective record-based cohort study design was conducted, targeting all accessible medical files of adult patients with SE who were admitted to the Aseer central hospital and military hospital from 2010 to 2017. Data were extracted from all complete and accessible files. Records with missing data were excluded. Clinical outcomes for the cases included were assessed and categorized into cases of complete recovery (without sequelae), cases with incomplete recovery, and death.

Results

The study included 19 adult patients with SE whose ages ranged from seven to 87 years with a mean age of 33.4 ± 22.5 years. Men made up 63.2% of the cases. Infection was the most recorded risk factor among the cases, followed by anti-epileptic drug withdrawal. Only two cases recovered with sequelae, while the remaining 17 cases recovered completely. There were no deaths.

Conclusions

The study revealed that nearly all cases recovered completely with no complications, particularly men who immediately received IV treatment. Early diagnosis and receiving treatment under careful observation via follow-up are recommended.

## Introduction

Epilepsy is a neurological disorder characterized by more than two unprovoked seizures [1]. Epileptic seizure episodes can vary from brief and nearly undetectable to long periods of vigorous shaking [2]. These episodes can result in physical injuries, occasionally including broken bones [3]. With epilepsy, seizures tend to recur and, as a rule, have no immediate underlying cause [4]. Single seizures occurring due to a specific cause, such as poisoning, are not considered epilepsy [5]. There are different treatments for epilepsy worldwide, and the condition carries varying degrees of social stigma [4]. Status epilepticus (SE) is an attack of an epileptic seizure lasting more than five minutes or two or more seizures without the person returning to normal between the attacks [6]. Previously, SE was defined by a 30-minute duration [7]. The seizures can be of different types, such as tonic-clonic, absence, or complex partial seizures [8]. It is a life-threatening emergency, especially if treatment is delayed [9]. SE can occur in patients diagnosed with epilepsy and those with brain pathology such as trauma, infections, or strokes [10]. Diagnosis of SE is made via checking the blood glucose levels, different modalities of imaging of the head, various blood tests, and an electroencephalogram [11]. Patients with psychogenic nonepileptic seizures may present similarly [12]. In addition, low blood glucose levels, movement disorders, meningitis, and delirium may also present similarly to SE. In Saudi Arabia, the prevalence rate for active epilepsy is 6.54 per 1000 population (95% CI of 5.48-7.60). Among these, 28% of the patients had partial seizures, 21% had generalized seizures; for the remaining 51%, it was not possible to determine if the generalized seizures had focal onset or not [13]. The current study aimed to assess patterns, correlations, and clinical outcomes of SE among patients with epilepsy in the Aseer region of Saudi Arabia.

## Materials and methods

A retrospective record-based cohort study design was conducted, targeting all accessible medical files of adult patients (aged ≥ 12 years) with SE who were admitted to the Aseer central hospital and military hospital from 2010 to 2017. Data were extracted from all complete and accessible files. Records with missing data were excluded. Data were extracted using a restructured data extraction format to avoid errors and reduce inter-rater bias. Data covered patients' demographic characteristics, including age, gender, and clinical and family history of seizure. Clinical data regarding SE were also collected, including the type of epilepsy, type of SE (whether it is generalized or partial), risk factors, and treatment received. Clinical outcomes for the included cases were assessed and categorized into cases with complete recovery (without sequelae), incomplete recovery, and death.

Data analysis

After data extraction, the data were reviewed, coded, and applied into software for statistical analysis (IBM SPSS Statistics for Windows, Version 22.0., IBM Corp., Armonk, NY). All statistical analyses were performed using a two-tailed test. A p-value of less than 0.05 was considered statistically significant. A descriptive analysis based on frequency and percent distribution was performed for all variables, including demographic data, epilepsy, and SE data. Due to the small frequencies, the univariant relations between patients' bio-demographic and clinical data with SE clinical outcomes were generated based on exact probability tests.

## Results

The study included 19 patients with SE whose ages ranged from seven to 87 years with a mean age of 33.4 ± 22.5 years. Men made up 63.2% of the cases, and 57.9% of the patients had reported concerns of primary epilepsy. Stroke and head trauma were the most recorded causes of secondary epilepsy (25% for each), followed by infection, tumor, and drugs (12.5% each). Eighteen cases (94.7%) had concerns of generalized tonic-clonic seizure, and 68.4% of the cases reported a first-time episode of SE (Table 1).

**Table 1:** Bio-demographic data of adult patients with status epilepticus in the Aseer region, Saudi Arabia. GTC: Generalized tonic-clonic; SE: Status epilepticus.

Bio-demographic data		N	%
Age	<30 years	11	57.9%
	>30 years	8	42.1%
Gender	Male	12	63.2%
	Female	7	36.8%
Type of epilepsy	Primary	11	57.9%
	Secondary	8	42.1%
If secondary, cause	Stroke	2	25.0%
	Infection	1	12.5%
	Tumor	2	25.0%
	Head trauma	1	12.5%
	Drug	1	12.5%
	Unknown	1	12.5%
Type of SE	GTC	18	94.7%
	Static partial seizure	1	5.3%
First-time admitted as SE	Yes	6	31.6%
	No	13	68.4%

**Figure 1:**
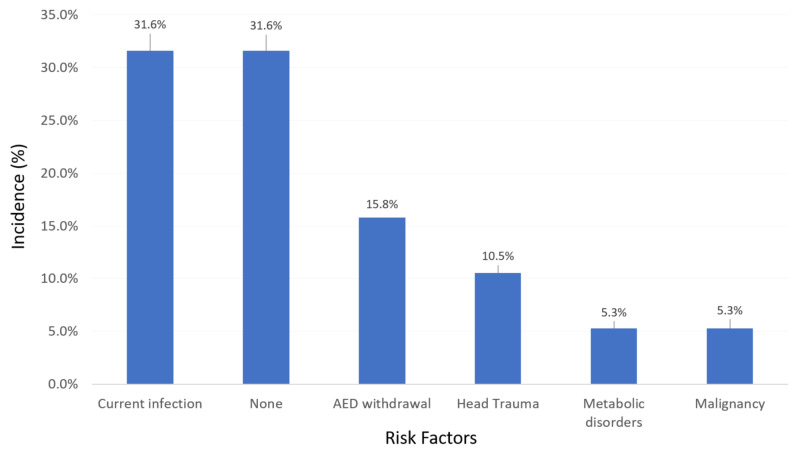
Risk factors of status epilepticus among adult patients with epilepsy in the Aseer region, Saudi Arabia. AED: Anti-epileptic drug.

Of the risk factors for SE recorded for the cases (Figure 1), infection was the most recorded (31.6%), followed by anti-epileptic drug (AED) withdrawal (15.8%), head trauma (10.5%), metabolic disorders (5.3%), and malignancy (5.3%).

Among the management methods used for these cases, fifteen cases (78.9%) received a benzodiazepine IV loading dose, followed by a phenytoin IV loading dose (73.7%), and Keppra IV loading dose (15.8%). Intubation was required in six cases (31.6%), while seven cases (36.8%) received anesthetic agents (midazolam [Dormicum] in two cases and fentanyl in three cases; three cases had both; Table 2).

**Table 2:** Management of status epilepticus among adult patients with epilepsy in the Aseer region, Saudi Arabia. IV: Intravenous; SE: Status epilepticus.

Management for SE	N	%
Benzodiazepine IV loading dose	15	78.9%
Phenytoin IV loading dose	14	73.7%
Valproate IV loading dose	1	5.3%
Keppra IV loading dose	3	15.8%
Intubation	6	31.6%
Anesthetic agents	7	36.8%
Midazolam only	2	28.6%
Fentanyl only	2	28.6%
Both	3	42.9%

For clinical outcomes, only two cases (10.5%) recovered with sequelae (focused neurological deficit), while the remaining 17 cases recovered completely (89.5%). There were no deaths. Upon comparing the clinical outcomes of each case with the case characteristics (Table 3), it was clear that all cases involving men had complete recovery compared to 71.4% for women; this difference reached significance (p = 0.048). Also, all cases that received a phenytoin IV loading dose had completely recovered compared to those who did not (p = 0.012). All other factors examined were not significantly related; a good recovery involved a young age, primary epilepsy, having other loading drugs, and anesthetic agents.

**Table 3:** Clinical outcome of adult patients with status epilepticus by their characteristics in Aseer region, Saudi Arabia. P: Exact probability test * P < 0.05 (significant) CPS: Complex partial seizures; GTC: Generalized tonic-clonic; SE: Status epilepticus.

Factors		Outcome				P-value
		Recovered with sequelae		Recovered without sequelae		
		N	%	N	%	
Age	<30 years	1	9.1%	10	90.9%	0.811
	> 30 years	1	12.5%	7	87.5%	
Gender	Male	0	0.0%	12	100.0%	0.048*
	Female	2	28.6%	5	71.4%	
Type of epilepsy	Primary	1	9.1%	10	90.9%	0.811
	Secondary	1	12.5%	7	87.5%	
Type of SE	GTC	2	11.1%	16	88.9%	0.725
	Status CPS	0	0.0%	0	0.0%	
	Static partial seizure	0	0.0%	1	100.0%	
First time admitted as SE	Yes	1	16.7%	5	83.3%	0.554
	No	1	7.7%	12	92.3%	
Benzodiazepine IV loading dose	Yes	2	13.3%	13	86.7%	0.440
	No	0	0.0%	4	100.0%	
Phenytoin IV loading dose	Yes	0	0.0%	14	100.0%	0.012*
	No	2	40.0%	3	60.0%	
Valproate IV loading dose	Yes	0	0.0%	1	100.0%	0.725
	No	2	11.1%	16	88.9%	
Keppra IV loading dose	Yes	0	0.0%	3	100.0%	0.517
	No	2	12.5%	14	87.5%	
Intubation	Yes	0	0.0%	6	100.0%	0.310
	No	2	15.4%	11	84.6%	
Anesthetic agents	Yes	0	0.0%	7	100.0%	0.253
	No	2	16.7%	10	83.3%	

## Discussion

SE is a common neurological emergency associated with a high mortality rate and poor outcomes. The duration of seizures defined in the diagnosis of SE has been shortened; it is now suggested to be five minutes to allow for earlier diagnosis and management. Recent reports found that the mortality rate of convulsive status epilepticus can reach up to 39% in some countries with poor outcomes [14]. Another study done in Brazil in 2015 found a case fatality rate of 36.2%, which was higher than in previous studies [15]. A third study performed in Thailand in August 2015 reported that 75.2% of patients improved while 16.4% did not and an in-hospital mortality rate of 8.4% [16]. However, the outcome varied by medical care setting and country.

The current study aimed to assess the pattern of SE recorded among these cases and to detect the distribution of risk factors for having SE. Additionally, the clinical outcome of these cases was a focus. This study revealed that most of the SE cases recovered completely without any neurological deficits or complications. The highest recovery rate was recorded among men who received phenytoin treatment. All other factors, including age, type of epilepsy or SE, and other drugs received, were not significantly related to better outcomes. This may be explained by the fact that only two cases had a poor recovery with sequelae, highlighting a need for a larger sample size to detect a significant difference. The most-reported risk factor for having an SE attack was an infection, followed by AED withdrawal and head trauma. These are all modifiable factors that can be managed to avoid attacks of SE among epilepsy cases.

These findings are consistent with that reported by Amare A et al. [17]. Their study was conducted on SE patients aged 13 years or older. The researchers concluded that a CNS infection was the most frequent factor behind SE and that AED withdrawal was the primary cause in patients with a history of epilepsy. Use of parenteral anticonvulsants, any emergency measurement of serum AED levels, and electroencephalography for diagnosis and monitoring were unavailable. Mortality was related to underlying etiologies, especially HIV/AIDS and its CNS complications.

Patients with epilepsy, particularly the young, may develop SE attacks that affect their quality of life and even end in death. Therefore, detection of these cases and mapping predictors of poor outcomes are important and one of the most important goals in these cases.

Limitations and strengths of the study

As the study was based on records from the two main hospitals in the southern region of Saudi Arabia, the sample size was very small. This led to a recommendation to prolong the duration of the study. However, this was a barrier for the researchers involved in the study as they were conducting it themselves and were not reimbursed. Also, many more important types of data would have been required for more precise conclusions, including seizures type, duration, and triggering and inhibitory factors. Despite these limitations, this study can raise awareness of these types of cases and give initial clues on how to handle these situations.

## Conclusions

Most SE cases completely recovered without any complications. No deaths were recorded. Male gender and having treatment promptly were the most important predictors of better outcomes. Early diagnosis and receiving treatment with close follow-up and control of seizures are recommended.

## References

[1] Epilepsy Fact Sheet, WHO WHO (2016). WHO, Epilepsy Fact Sheet. 2016. Retrieved.

[2] Goldberg EM, Coulter DA (2013). Mechanisms of epileptogenesis: a convergence on neural circuit dysfunction. Nat Rev Neurosci.

[3] Chang BS, Lowenstein DH (2003). Epilepsy. N Engl J Med.

[4] Fisher RS, Acevedo C, Arzimanoglou A (2014). ILAE official report: a practical clinical definition of epilepsy. Epilepsia.

[5] Fisher RS, van Emde Boas W, Blume W, Elger C, Genton P, Lee P, Engel J Jr (2005). Epileptic seizures and epilepsy: definitions proposed by the International League Against Epilepsy (ILAE) and the International Bureau for Epilepsy (IBE). Epilepsia.

[6] Al-Mufti F, Claassen J (2014). Neurocritical care: status epilepticus review. Crit Care Clin.

[7] Trinka E, Höfler J, Zerbs A (2012). Causes of status epilepticus. Epilepsia.

[8] Prasad M, Krishnan PR, Sequeira R, Al-Roomi K (2014). Anticonvulsant therapy for status epilepticus. Cochrane Database Syst Rev.

[9] Rossetti AO, Lowenstein DH (2011). Management of refractory status epilepticus in adults: still more questions than answers. Lancet Neurol.

[10] Stasiukyniene V, Pilvinis V, Reingardiene D, Janauskaite L (2009). [Epileptic seizures in critically ill patients]. Medicina (Kaunas).

[11] Nair PP, Kalita J, Misra UK (2011). Status epilepticus: why, what, and how. J Postgrad Med.

[12] Rudin D, Grize L, Schindler C, Marsch S, Rüegg S, Sutter R (2011). High prevalence of nonconvulsive and subtle status epilepticus in an ICU of a tertiary care center: a three-year observational cohort study. Epilepsy Res.

[13] Al Rajeh S, Awada A, Bademosi O, Ogunniyi A (2001). The prevalence of epilepsy and other seizure disorders in an Arab population: a community-based study. Seizure.

[14] Leitinger M, Kalss G, Rohracher A (2015). Predicting outcome of status epilepticus. Epilepsy Behav.

[15] Stelzer FG, Bustamante GO, Sander H, Sakamoto AC, Fernandes RM (2015). Short-term mortality and prognostic factors related to status epilepticus. Arq Neuropsiquiatr.

[16] Tiamkao S, Pranboon S, Thepsuthammarat K, Sawanyawisuth K (2015). Incidences and outcomes of status epilepticus: a 9-year longitudinal national study. Epilepsy Behav.

[17] Amare A, Zenebe G, Hammack J, Davey G (2008). Status epilepticus: clinical presentation, cause, outcome, and predictors of death in 119 Ethiopian patients. Epilepsia.

